# Comprehensive Assessment and Early Prediction of Gross Motor Performance in Toddlers With Graph Convolutional Networks–Based Deep Learning: Development and Validation Study

**DOI:** 10.2196/51996

**Published:** 2024-02-21

**Authors:** Sulim Chun, Sooyoung Jang, Jin Yong Kim, Chanyoung Ko, JooHyun Lee, JaeSeong Hong, Yu Rang Park

**Affiliations:** 1 Department of Biomedical Systems Informatics Yonsei University College of Medicine Seoul Republic of Korea

**Keywords:** child development, digital health, artificial intelligence, gross, motor, movement, development, developmental, machine learning, pediatric, pediatrics, paediatric, paediatrics, toddler, toddlers, child, children, limb, limbs, algorithm, algorithms, kinesiology, GCN, graph convolutional networks, convolutional network

## Abstract

**Background:**

Accurate and timely assessment of children’s developmental status is crucial for early diagnosis and intervention. More accurate and automated developmental assessments are essential due to the lack of trained health care providers and imprecise parental reporting. In various areas of development, gross motor development in toddlers is known to be predictive of subsequent childhood developments.

**Objective:**

The purpose of this study was to develop a model to assess gross motor behavior and integrate the results to determine the overall gross motor status of toddlers. This study also aimed to identify behaviors that are important in the assessment of overall gross motor skills and detect critical moments and important body parts for the assessment of each behavior.

**Methods:**

We used behavioral videos of toddlers aged 18-35 months. To assess gross motor development, we selected 4 behaviors (climb up the stairs, go down the stairs, throw the ball, and stand on 1 foot) that have been validated with the Korean Developmental Screening Test for Infants and Children. In the child behavior videos, we estimated each child’s position as a bounding box and extracted human keypoints within the box. In the first stage, the videos with the extracted human keypoints of each behavior were evaluated separately using a graph convolutional networks (GCN)–based algorithm. The probability values obtained for each label in the first-stage model were used as input for the second-stage model, the extreme gradient boosting (XGBoost) algorithm, to predict the overall gross motor status. For interpretability, we used gradient-weighted class activation mapping (Grad-CAM) to identify important moments and relevant body parts during the movements. The Shapley additive explanations method was used for the assessment of variable importance, to determine the movements that contributed the most to the overall developmental assessment.

**Results:**

Behavioral videos of 4 gross motor skills were collected from 147 children, resulting in a total of 2395 videos. The stage-1 GCN model to evaluate each behavior had an area under the receiver operating characteristic curve (AUROC) of 0.79 to 0.90. Keypoint-mapping Grad-CAM visualization identified important moments in each behavior and differences in important body parts. The stage-2 XGBoost model to assess the overall gross motor status had an AUROC of 0.90. Among the 4 behaviors, “go down the stairs” contributed the most to the overall developmental assessment.

**Conclusions:**

Using movement videos of toddlers aged 18-35 months, we developed objective and automated models to evaluate each behavior and assess each child’s overall gross motor performance. We identified the important behaviors for assessing gross motor performance and developed methods to recognize important moments and body parts while evaluating gross motor performance.

## Introduction

For the continuous and proper development of children, an accurate and timely assessment of their developmental levels is essential [1]. Early diagnosis during the toddler stage allows for early intervention, which can significantly impact children’s later life outcomes [2,3]. Previous research has shown that early intervention in vulnerable populations, such as those with low birth weight and prematurity, leads to significant improvements in later childhood developments compared to those who do not receive early intervention and that these differences persist into adolescence [4,5]. Numerous studies have shown that the influence of early intervention extends beyond adolescence to adulthood, with significant socioeconomic benefits [6-8]. A recent study about the development of children exposed to lead showed that early intervention before the age of 3 years benefited their future academic performance [9].

As a result, many countries recommend the need for regular developmental screening of infants and young children, and South Korea has implemented the National Health Screening Program for Infants and Children for children under 6 years of age since 2007 [10-14]. The National Health Screening Program for Infants and Children in South Korea developed the Korean Developmental Screening Test for Infants and Children (K-DST) in 2014, which assesses gross motor, fine motor, cognitive, language, social, and self-help skills in children aged 4-71 months [15].

In various areas of development, gross motor development begins earlier than other areas of development, such as fine motor and language development, and therefore, it is possible to assess the risk of developmental delay at a younger age by monitoring gross motor development. Studies have shown that gross motor development at an early age is predictive of subsequent developments [16] and is also associated with future academic achievement [17,18].

However, a global shortage of pediatric health care providers hinders the proper developmental assessment of children. In a report published in 2016, one-third of pediatricians in the United States did not use standardized screening tools in their pediatric practice because of issues such as limited clinic hours and a shortage of medical staff to perform developmental screenings [19]. As an alternative to pediatric health care professionals, many countries, including South Korea, rely on parental reports to determine developmental milestones. However, parental reports are based on subjective opinions, and parents may respond positively even when they have observed their child’s activities only once, leading to false positives [20]. Based on these factors, there is a need for an objective, labor-free, and automated tool to assess the development of children.

Recently, there have been several studies using deep learning to assess gross motor development in children. A study reported that a deep learning model can predict cerebral palsy progression from videos of spontaneous movements taken in infancy [21]. However, this study did not use a previously validated metric such as the K-DST, which may limit the explainability and generalizability of the model. Liu et al [22] evaluated the gross motor skills of children with autism with an average age of 5 years, and Suzuki et al [23,24] assessed gross motor skills on a video-by-video basis using a deep learning model with behavioral videos of 4- to 5-year-old children. However, since these studies were conducted on children aged ≥4 years, there is a limitation in that they could not validate the model effectiveness in the <3 years age group, where early intervention is expected to be more effective.

Considering these factors, we developed an automated and accurate pediatric developmental assessment model using videos of toddlers aged 18-35 months performing gross motor movements that have been validated with the K-DST. Our 2-step model assesses each behavior and evaluates each child’s overall gross motor performance based on the performance level of each behavior. In addition, we identified behaviors that contribute to the overall gross motor skills assessment and detected critical moments and important body parts for the assessment of each behavior.

## Methods

### Study Design and Participants

In this study, we used behavioral videos of toddlers aged 18-35 months, when most of them could walk, perform a wide range of gross motor actions, and minimally understand the examiner’s instructions to perform the task [16,25]. We selected 4 behaviors frequently used by the K-DST to assess gross motor development in this age group: climb up the stairs, go down the stairs, throw the ball, and stand on 1 foot [15]. These 4 movements were chosen as core tasks based on existing child development guidelines and in consultation with 3 pediatricians and 15 child development experts, considering the physical and cognitive abilities of this age group [26,27]. The participant performed multiple trials for each behavior. For each of these trials, the raters watched the video and rated the performance as “bad,” “good,” or “perfect.”

We also categorized participants into “relatively slow” and “relatively fast” groups based on their overall performance: if their performance was rated as “bad” on 2 or more behaviors, we categorized them as “relatively slow”; the remaining cases were categorized as “relatively fast.”

### Ethical Considerations

The data set used in this study is from our previous study and it was constructed while adhering to the ethical principles of the Declaration of Helsinki [28]. The construction of the data set was approved by the Institutional Review Board of Severance Hospital, Yonsei University College of Medicine (4-2021-0845), and the requirement for informed consent was waived due to the retrospective nature of the study. Participants of the data set were recruited from daycare centers, kindergartens, primary pediatric hospitals, and internet communities. Written informed consent for data collection and subsequent analysis was obtained from all caregivers of the participants. Participants received ₩50,000 (approximately US $38) and were provided with an intelligence scale test valued at around ₩300,000 (US $232) as compensation. To ensure the confidentiality and privacy of the participants, each study participant was deidentified via an alphanumeric code.

### Experimental Setting

The videos were recorded in the presence of caregivers, examiners, and children. Depending on the behavior, a staircase or a ball was used as the apparatus. The video recordings for each child were conducted for approximately 1 hour. A camera was positioned to capture the entire body of each child. Using a frontal angle camera, the child’s behavior was recorded as an RGB (red-green-blue) video. All videos were collected using a Sony DSC-RX100 with 1920×1080 resolution and at 30 frames per second. The collected videos were rated by human raters based on the K-DST criteria, and these values were used as true labels in the stage-1 model.

### Data Preprocessing

To assess the behavior of the children in the RGB videos, we estimated the position of each child as a bounding box and then extracted 17 human keypoints within the box [29]. To detect the participants, we estimated the bounding boxes using Faster-RCNN [30] with the ResNet 50 backbone in the RGB videos. HRNet was then used to detect human keypoints in the detected bounding boxes [31]. Skeleton data were generated at a rate of 30 frames per second.

### Model Construction

**Figure 1:**
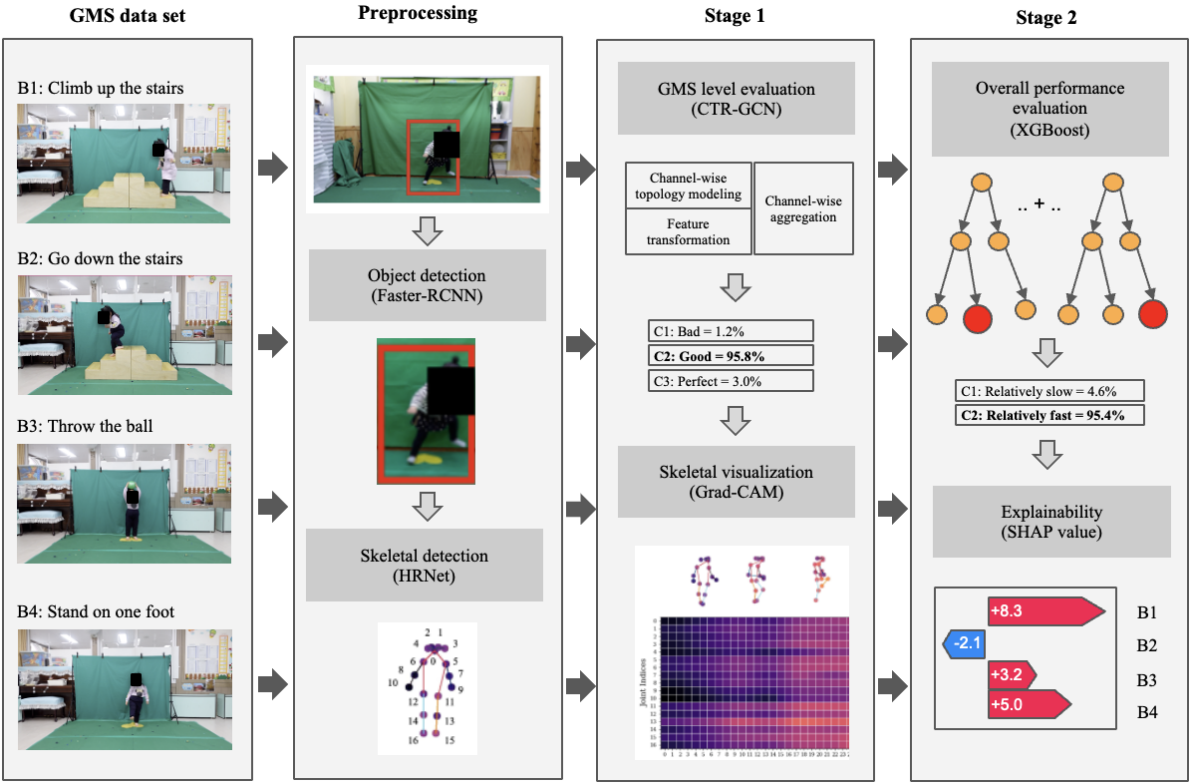
An overview of the suggested 2-stage model for predicting and evaluating comprehensive gross motor performance of children. Faster-RCNN and HRNet were used to extract the skeletal joints from the 4 behavioral videos. The evaluation of each behavior in the stage-1 was performed by graph convolutional networks model separately, and Grad-CAM was used for analyzing the influence of each joint and time segment of the video. In stage-2, the XGBoost algorithm was used for overall performance evaluation, and the SHAP method was used to recognize the contribution of each behavior to the evaluation. B: behavior; C: class; CTR-GCN: channel-wise topology refinement graph convolution networks; Grad-CAM: gradient weighted class activation mapping; GMS: gross motor skills; SHAP: Shapley additive explanations; XGBoost: extreme gradient boosting.

**Figure 2:**
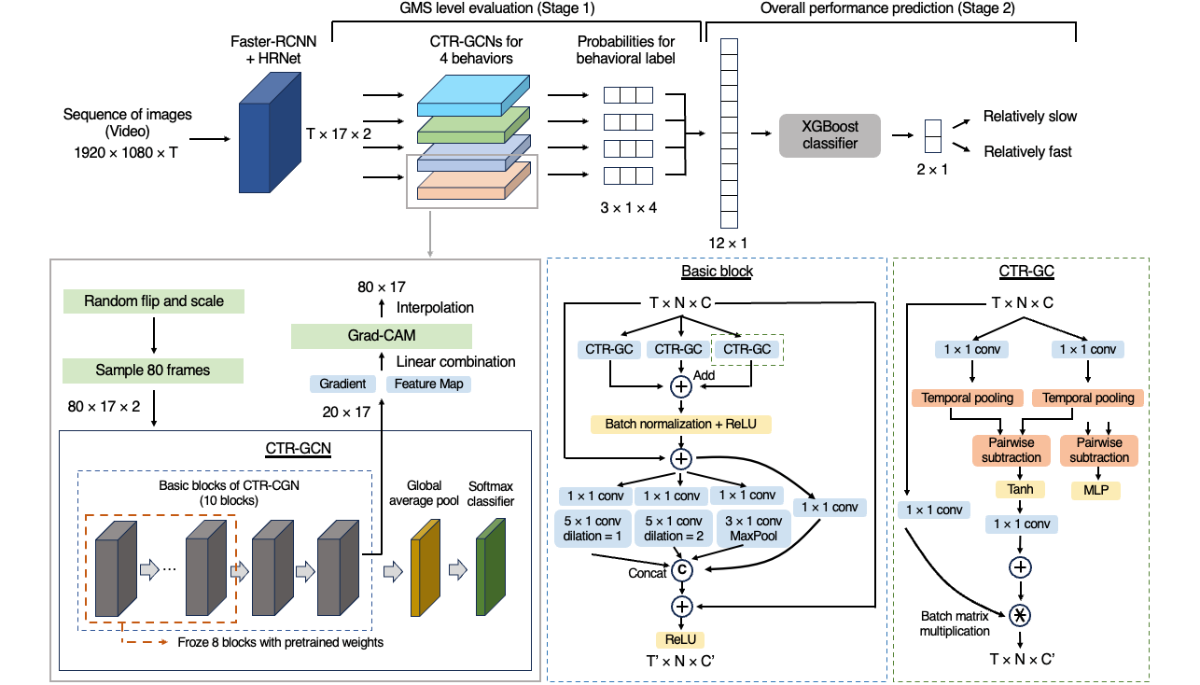
A detailed architecture of the suggested model and CTR-GCN. We applied augmentation methods and frame sampling strategies. The pretrained weights for the CTR-GCN model were applied, and 8 basic blocks of the model were frozen during training. The Grad-CAM was generated from the gradients and feature map from the last block. The Grad-CAM was then interpolated to align with the input frames. A basic block of CTR-GCN consists of 3 CTR-GCs, which use temporal pooling to aggregate temporal features of skeleton graph sequences and pairwise subtraction and concatenation for correlation modeling between skeletal joints. C: channel dimension of the data; CTR-GCN: channel-wise topology refinement graph convolution networks; GMS: gross motor skills; Grad-CAM: gradient-weighted class activation mapping; MLP: multilayer perceptron; N: number of skeletal joints; T: temporal dimension of the data; Tanh: hyperbolic tangent function; XGBoost: extreme gradient boosting.

We divided the data into training, validation, and test sets in a 6:2:2 ratio for each behavior, ensuring that data from the same individual were not allocated across multiple sets. To predict the overall gross motor performance of the children, we designed a 2-stage model. The overview of our model is shown in Figure 1. The first stage is the action evaluation stage, in which each behavior is evaluated separately using a graph convolutional networks (GCN)–based deep learning algorithm. To improve the performance of the stage-1 model, we performed transfer learning with pretrained weights. These pretrained weights are released by PYSKL and are trained with the channel-wise topology refinement graph convolution networks (CTR-GCN) model on the NTU RGB+D dataset by detecting 17 skeleton nodes with HRNet [32-34]. The CTR-GCN model is a stacked structure of 10 basic blocks, 8 of which were frozen during the training on our data. Augmentation using random flipping and scaling was applied to our training data. The training task was repeated 5 times for the same data, and 80 frames were randomly selected each time. The model training strategy of this study and the architecture of the CTR-GCN is shown in Figure 2. A total of 4 CTR-GCN models were trained to generate the predicted probabilities for the 4 gross motor skills, 1 for each behavior [35]. Although these 4 models can assess the performance of each behavior, it was necessary to integrate all 4 models to have a comprehensive assessment of the child’s gross motor development. Accordingly, to assess overall gross motor performance, the stage-2 model aggregated the outcome probability values of each label per behavior. The extreme gradient boosting (XGBoost) algorithm was used for the stage-2 model [36]. The validation process was performed using a 10-fold cross-validation strategy. The parameters used to train our models are shown in Multimedia Appendix 1.

### Evaluation of Model Performance and Verification of Explainability

The stage-2 model–assessed performance was compared with human panel–assessed performance on a fixed-test data set. A panel consisting of 1 pediatrician and 2 nonexperts assessed the participants’ overall gross motor status. Sensitivity and specificity for each panel were calculated.

For the interpretability of the stage-1 action evaluation model, gradient-weighted class activation mapping (Grad-CAM) was used to identify critical time points and body parts in behavioral videos [37]. To create a Grad-CAM heatmap, we obtained weights for each label through gradient calculation and extracted feature maps from the final graph convolutional layer of the CTR-GCN model. The heatmap was then generated by linearly combining the derived weights and feature maps and applying the ReLU function [38]. We then visualized the heatmap along with the original input, which is a sequence of positions of skeletal joints. To understand the influence of each body part on model decision, we determined the top-1 activated joints that had the highest Grad-CAM values per frame and grouped their frequency by body part [39]. The 17 joints were grouped as belonging to the head, left arm, right arm, left leg, and right leg [39].

In the second stage of overall performance prediction, we used the Shapley additive explanations (SHAP) method to identify the actions that contribute more to the total developmental assessment [40]. The mean absolute SHAP value was obtained to estimate the contribution of each feature to the model output.

### Statistical Analysis

The performance of the models was evaluated using the area under the receiver operating characteristic curve (AUROC) score, which was calculated as the average of all folds and presented with SD. The optimal cutoff value for the overall gross motor skill assessment was determined based on receiver operating characteristic analysis using the Youden index. The receiver operating characteristic curve was also plotted with the average of the folds within the threshold intervals and the area between the SDs. All statistical analyses were performed in Python (version 3.6.8; Python Software Foundation) using *sci-kit-learn* (0.24.2 version).

## Results

### Characteristics of Cohort Participants

Behavioral videos of the 4 gross motor skills were collected from 141 children, of which 71 (50.4%) were boys, and 70 (49.6%) were girls. The average age of the children in this study was 29.6 (SD 4.3) months. The characteristics of the behavioral data for each gross motor skills are listed in Table 1. A total of 2502 behavioral videos were collected, with 698 (23.9%) rated as “bad,” 581 (23.2%) rated as “good,” and 1321 (52.8%) rated as “perfect.”

**Table 1:** Characteristics of cohort participants. The 141 participants consisted of 71 (50.4%) boys and 70 (49.6%) girls, and the average age was 29.6 (SD 4.3) months. A total of 2502 behavioral videos were collected, with 698 (23.9%) rated as “bad,” 581 (23.2%) rated as “good,” and 1321 (52.8%) rated as “perfect.” The distribution of the demographics of the population and the number of videos by label for each behavior are represented as the total number and its percentage.

Parameter and variable			Type of gross motor skill				
			Climb up the stairs (N=141)	Go down the stairs (N=141)	Throw the ball (n=140)	Stand on 1 foot (N=141)	Total (N=141)
**Demographics**							
	Age (months), mean (SD)		29.5 (4.3)	29.5 (4.3)	29.5 (4.3)	29.5 (4.3)	28.6 (4.3)
	**Gender, n (%)**						
		Girls	70 (49.6)	70 (49.3)	69 (49.3)	70 (49.6)	70 (49.6)
		Boys	71 (50.4)	71 (50.7)	71 (50.4)	71 (50.4)	71 (50.4)
**Number of videos by label, n (%)**							
	Bad		137 (21.9)	144 (23.1)	95 (15.0)	222 (35.9)	598 (23.9)
	Good		106 (16.9)	107 (17.2)	156 (24.7)	212 (34.2)	581 (23.2)
	Perfect		384 (61.2)	372 (59.7)	380 (60.2)	185 (29.9)	1321 (52.8)

### Performance of the Evaluation of Each of the 4 Gross Motor Skills

**Figure 3:**
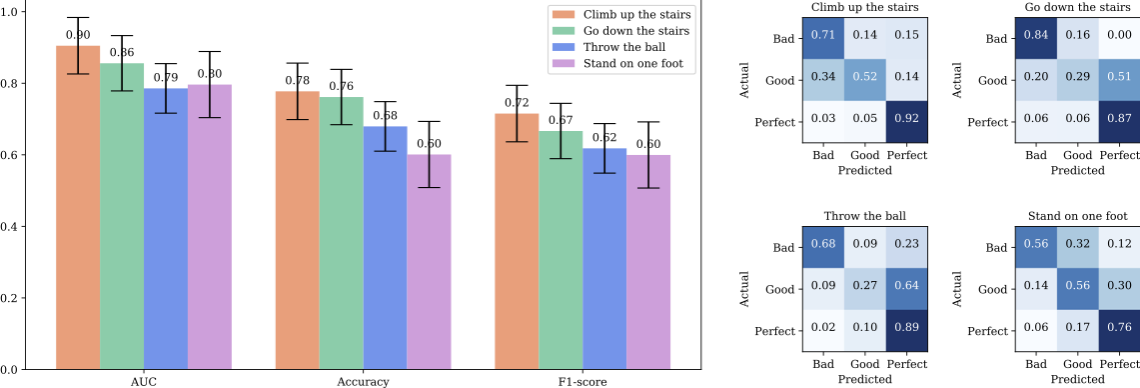
Performance scores and confusion matrices of the stage-1 gross motor skill evaluation model for 4 behaviors. The bar chart of scores is shown with error bars indicating the range between minimum and maximum scores observed in the cross-validation. The AUC scores range from 0.79 for “throw the ball” to 0.90 for “climb up the stairs.” The confusion matrices show the model’s ability to distinguish between “bad” and “good” labels. AUC: area under the curve.

Table 2 shows the results of the first-stage model. The AUROC values with each behavioral evaluation were from 0.79 to 0.90. We found that the model for the “climb up the stairs” behavior performed the best, with an AUROC score of 0.90, followed by “go down the stairs” with an AUROC score of 0.86; subsequently, the models for “throw the ball” and “stand on 1 foot” performed similarly, with AUROC scores of 0.79 and 0.80, respectively (Figure 3).

The model generally made good predictions for both “bad” and “perfect” behaviors when we calculated the normalized confusion matrix for all behaviors but tended to struggle to distinguish “good” behaviors that represent the intermediate stage (Figure 3). Specifically, for the “go down the stairs” behavior, the model accurately predicted the “bad” behavior with a ratio of 0.84. Even when the prediction was incorrect, the model tended to classify the behavior as “good,” which is the adjacent label to “bad.”

**Table 2:** Results of the evaluation of the 4 gross motor skills.

Performance metric	Gross motor skill, mean (SD)			
	Climb up the stairs	Go down the stairs	Throw the ball	Stand on 1 foot
Accuracy	0.78 (0.02)	0.76 (0.03)	0.68 (0.02)	0.60 (0.02)
Sensitivity	0.71 (0.03)	0.67 (0.03)	0.61 (0.02)	0.63 (0.02)
Specificity	0.86 (0.02)	0.85 (0.02)	0.78 (0.01)	0.76 (0.02)
*F*_1_-score	0.72 (0.04)	0.67 (0.04)	0.62 (0.03)	0.60 (0.03)
AUROC^a^	0.90 (0.01)	0.86 (0.02)	0.79 (0.02)	0.80 (0.02)

^a^AUROC: area under the receiver operating characteristic curve.

### Grad-CAM on the Visualization of Human Keypoint

**Figure 4:**
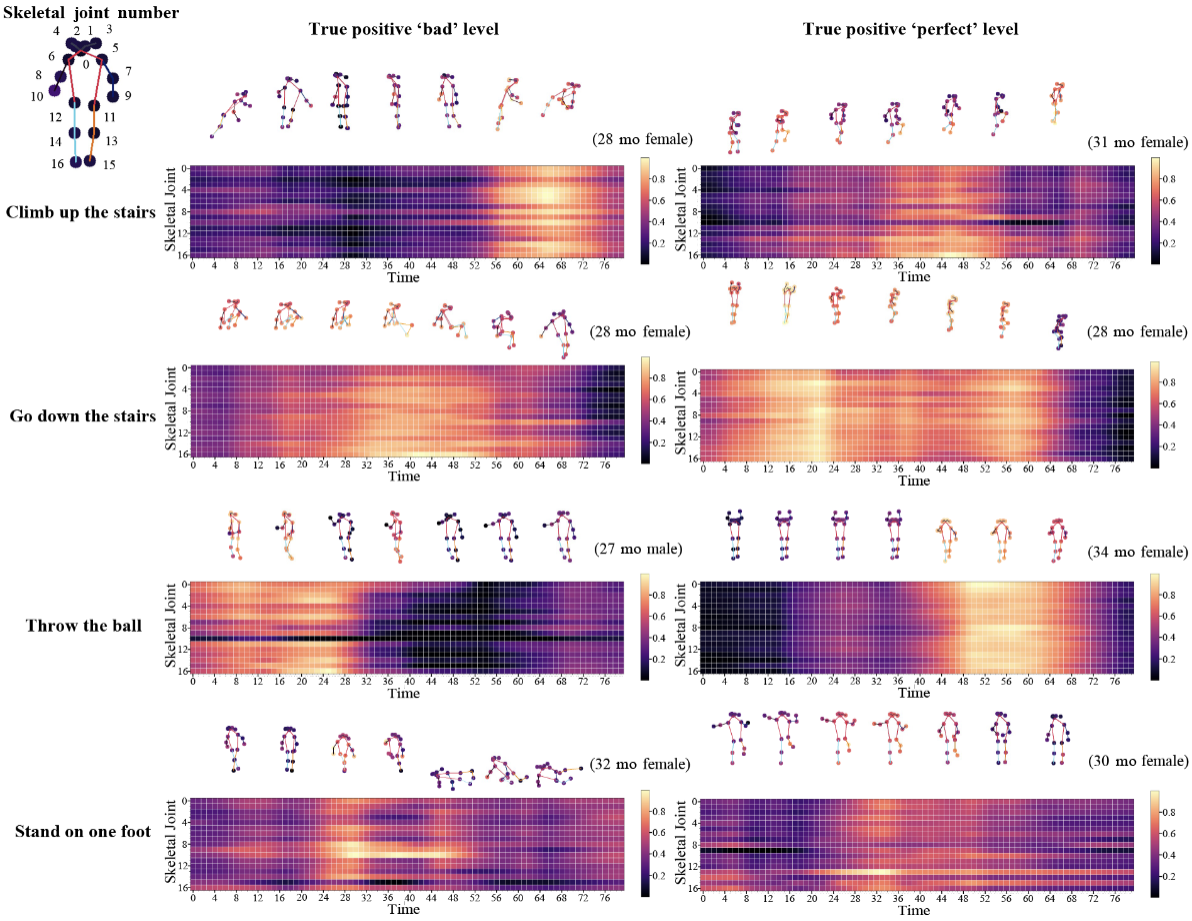
Grad-CAM heatmap with frame-by-frame mapped keypoints for each behavior. The change of Grad-CAM values over time for 17 human keypoints was displayed as a heatmap. For each behavior, the Grad-CAM heatmap for a given participant was compared between a “perfect” and “bad” performance. The actions of the participant over time were visualized as human keypoints and shown above the heatmap. The age and gender of each child were displayed together. Grad-CAM: gradient-weighted class activation mapping.

Our keypoint-mapping Grad-CAM visualization showed the differences in the activated joints for each behavior and label (Figure 4). By observing the highlighted areas in the heatmap, we could identify the contribution of the joints to the evaluation of each behavior. The horizontal axis, labeled as “time,” indicates the moments of the behavioral video that contributed to the classification across the selected 80 frames of the videos. The vertical axis, labeled as “skeletal joint,” shows the critical joints related with behavior evaluation. For the “climb up the stairs” behavior, the Grad-CAM results of the behavior evaluated as “bad” showed that the Grad-CAM scores of the arms and head increased as the child falls and grabs the stairs with their hands. On the other hand, for behavior evaluated as “perfect,” the child’s legs scored consistently high as they walked up.

It was also observed that the Grad-CAM score was higher when a given task was being performed. In the Grad-CAM results for the child who was rated “bad” for “climb up the stairs,” we could observe that the moment when the child wandered and looked back to the assistant has a lower Grad-CAM value than the moment when they climbed the stairs. To compare the importance of each body part, we determined the top-1 activated joint, which is the joints with the highest Grad-CAM value per frame, and grouped the frequencies by body part. (Multimedia Appendix 2) [39].

### Overall Performance Status Prediction

**Figure 5:**
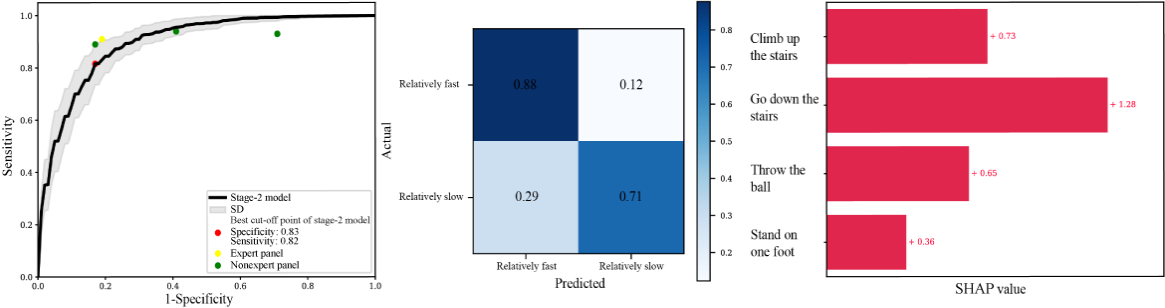
ROC curves, confusion matrix, and grouped SHAP values of the stage-2 overall performance status prediction model. The stage-2 overall performance status prediction model had an AUC score of 0.90, and the specificity and sensitivity of the optimal cutoff points were 0.83 and 0.82, respectively. For the expert panel, the sensitivity and specificity were 0.91 and 0.81, respectively. For the nonexpert panels, the mean sensitivity and mean specificity were 0.90 and 0.59, respectively. In the confusion matrix, we displayed the relative ratio of the predicted values to each actual value. To identify the highly contributed behaviors in the stage-2 overall performance status prediction model, we obtained the SHAP value of each label in 4 behaviors and summed the SHAP values for each behavior. AUC: area under the curve; ROC: receiver operating characteristic; SHAP: Shapley additive explanations.

The results of the stage-2 overall performance prediction model and the human panels on a fixed-test data set are shown in Figure 5. The model had an AUC score of 0.90, and the specificity and sensitivity of the optimal cutoff points were 0.83 and 0.82, respectively. For the human panels, sensitivities of 0.90 and 0.91 and specificities of 0.59 and 0.81 were recorded by nonexperts and an expert, respectively. Comparing each of these showed that the model performed better than the nonexpert panel and was similar to the expert panel. Table 3 shows the overall results of the model.

According to the grouped SHAP value obtained from variables for each action, the action “go down the stairs” contributed the most to the prediction, with a SHAP value of 1.28 (Figure 5). The next highest values were “climb up the stairs,” “throw the ball,” and “stand on 1 foot,” with values of 0.73, 0.65, and 0.36, respectively.

**Table 3:** Results of the stage-2 overall performance status prediction model.

Performance metric	Performance, mean (SD)
Accuracy	0.82 (0.04)
Sensitivity	0.71 (0.09)
Specificity	0.88 (0.04)
*F*_1_-score	0.74 (0.06)
AUROC^a^	0.90 (0.02)

^a^AUROC: area under the receiver operating characteristic curve.

## Discussion

### Principal Findings

In this study, we evaluated each gross motor behavior and assessed each child’s overall gross motor performance status using movement videos of toddlers aged 18-35 months. To the best of our knowledge, this study is the first to predict the overall gross motor behavior status using pediatric gross motor movement videos at ages younger than 3 years.

Several previous studies have attempted to predict pediatric development using digital phenotype data, such as detecting developmental disabilities using drag-and-drop data in games [41], identifying visual impairments using gaze patterns and facial feature data in response to visual stimuli on a smartphone, and measuring fine motor skills in children using sensor-augmented toys [42]. Suzuki et al [23,24] conducted studies that collected the behavioral videos of 4- to 5-year-old children and extracted skeletal data through OpenPose to evaluate behavioral performance on a per-video basis using a convolutional neural network and autoencoder model. Liu et al [22] proposed a method to evaluate the initial gross motor skills of children with autism with an average age of 5 years using velocities, trajectories, and angles of upper and lower limb joints based on skeleton data extracted through OpenPose. However, unlike these previous studies of gross motor skill assessments, our study focused on gross motor function in toddlers younger than the age of 3 years, which may allow us to quickly identify developmental delays in children younger than the age of 3 years for early intervention. Additionally, this study not only assessed each behavior but also built a model to evaluate the overall performance of each individual by aggregating the assessments of each behavior.

In this work, we performed action recognition using CTR-GCN on skeleton data extracted through human pose estimation with Faster-RCNN and HRNet [30,31,35]. Recently, many studies have been published on action recognition, which is broadly categorized into RGB-based methods and skeleton-based methods [43]. In this study, instead of RGB-based methods, which directly use RGB video, we used a skeleton-based method using Faster-RCNN and HRNet to estimate the location of human presence as a bounding box and extract human keypoints [30,31]. These skeleton-based methods are not only computationally efficient but also have the advantage of focusing on the child’s behavior and deidentifying the study participants by removing background information [43,44].

For human pose estimation, we used HRNet and Faster-RCNN compared to the studies by Suzuki et al [23,24] and Liu et al [22], which used OpenPose [30,31,45]. In human pose estimation, there are 2 types of methods: the bottom-up method (eg, OpenPose), where each body part is detected first and subsequently the body parts are combined, and the top-down method (eg, HRNet + Faster-RCNN), where the person is detected and then each body part is searched within the detected bounding box [29-31,45]. The HRNet method is known to be more accurate than OpenPose, and the top-down method is expected to be more accurate in detecting body parts, especially when there are multiple people in the video [31,32]. Since children are often filmed with their caregivers in the developmental test, the HRNet was more suitable for our study.

The types of behaviors assessed in this study have been used in the K-DST for the corresponding age group, and previous research has shown that these types of gross motor behaviors are good predictors of childhood developmental disorders, such as intellectual disability, autism spectrum disorder, and cerebral palsy [15]. Furthermore, the model we developed in this study provides more objective assessments of gross motor skills than the K-DST, which relies on parental reports and enables the assessment of gross motor skills to be automated without requiring trained pediatric health care providers.

Of the 4 behaviors evaluated, “go up the stairs” was the most accurately classified; however, in the actual model, “go down the stairs” had a higher contribution in SHAP values (Figures 2 and 4). When viewing videos of actual children’s behaviors, we found that while performing the “go down the stairs” behavior, the examiner placed the child on the stairs, and the child subsequently performed the action of going down the stairs to return to the caregiver at the bottom of the stairs without the examiner’s intervention. Other behaviors required frequent intervention by the investigator to encourage the child to perform the behavior successfully, because the children sometimes did not understand the investigator’s instructions (eg, holding up 1 leg for more than 1 second) or had a variety of alternative actions at the onset of the behavior (eg, returning to the caregiver instead of climbing the stairs).

We also aimed to validate the explainability of the model by calculating the Grad-CAM values of each joint for each behavior, frame by frame (Figure 4). This allowed us to identify specific joints that had high importance values at critical points in the child’s behavior. For example, in a video of a child performing the “stand on 1 foot” behavior, when we analyzed the Grad-CAM of each joint on a frame-by-frame basis, we could observe that the importance of the leg joints increased as the child stood on 1 leg. The importance of each joint across the videos was determined by counting the number of times each joint was the most important in a particular frame (Multimedia Appendix 2) [39]. This allowed us to identify the vital body parts for evaluating each behavior. In the case of “climb up the stairs,” for example, it was found that the values in the arm area increased when the child was performing the behavior poorly. This finding can be attributed to the child’s tendency to resort to crawling instead of standing when the child had difficulty climbing, thereby increasing the values in the arm. The analysis of Grad-CAM values per joint in the children’s behavioral videos allowed us to identify which joints were important for certain behaviors and which body parts were more deficient in each child during specific behaviors.

One limitation of this study was that we could not validate the model’s performance in different patient populations. The study used data from participants aged 18-35 months, as this is the developmental stage when children can perform a wide range of gross motor movements, such as walking and running, and can understand simple verbal instructions from the examiner. Therefore, further research is needed to determine which gross motor activities in different age groups can be used to assess gross motor development in children. In addition, because this study was limited to Korean children, we suggest that its applicability should be studied in various settings, including other ethnicities and cultural settings.

Additionally, this study did not collect long-term follow-up prognostic data on the participants, such as the subsequent occurrence of developmental delays. If prospective data had been collected on the occurrence of future developmental disabilities (eg, cerebral palsy and autism spectrum disorders), more thorough studies could have been conducted using our model. Therefore, it is necessary to consider the long-term prognosis follow-up of participants in future studies.

### Conclusions

We developed a model to assess 4 behaviors using behavioral video in children aged 18-35 months and to assess each child’s overall gross motor performance. This is the first study to assess the overall gross motor behavioral status of children younger than 3 years of age using gross motor video for automated and objective prediction of child development. We also identified important behaviors during the model’s assessment of overall gross motor performance. Furthermore, we developed a method to identify important moments and key body parts during behavioral assessment using Grad-CAM. We anticipate that a more accurate and automated assessment of gross motor development will be possible with this model if more data are available in a variety of settings.

## Appendix

Multimedia Appendix 1 Parameters used to train the model.

Multimedia Appendix 2 Distribution of top-1 activated joints per frame grouped by body parts.

## Abbreviations

AUROC: area under the receiver operating characteristic curve
CTR-GCN: channel-wise topology refinement graph convolution networks
GCN: graph convolutional networks
Grad-CAM: gradient-weighted class activation mapping
K-DST: Korean Developmental Screening Test for Infants and Children
RGB: red-green-blue
SHAP: Shapley additive explanations
XGBoost: extreme gradient boosting

## Data Availability

We used publicly available data from our previous research [28]. These data are available on GitHub [46].

## References

[1] Dworkin PH (1993). Detection of behavioral, developmental, and psychosocial problems in pediatric primary care practice. Curr Opin Pediatr.

[2] Ramey CT, Ramey SL (1998). Prevention of intellectual disabilities: early interventions to improve cognitive development. Prev Med.

[3] Ramey CT, Ramey SL (2004). Early learning and school readiness: can early intervention make a difference?. Merrill Palmer Q (Wayne State Univ Press).

[4] McCarton CM, Brooks-Gunn J, Wallace IF, Bauer CR, Bennett FC, Bernbaum JC, Broyles RS, Casey PH, McCormick MC, Scott DT, Tyson J, Tonascia J, Meinert CL (1997). Results at age 8 years of early intervention for low-birth-weight premature infants. the Infant Health and Development Program. JAMA.

[5] McCormick MC, Brooks-Gunn J, Buka SL, Goldman J, Yu J, Salganik M, Scott DT, Bennett FC, Kay LL, Bernbaum JC, Bauer CR, Martin C, Woods ER, Martin A, Casey PH (2006). Early intervention in low birth weight premature infants: results at 18 years of age for the Infant Health and Development Program. Pediatrics.

[6] Adams RC, Tapia C, Council on Children With Disabilities (2013). Early intervention, IDEA Part C services, and the medical home: collaboration for best practice and best outcomes. Pediatrics.

[7] Herrod HG (2007). Do first years really last a lifetime?. Clin Pediatr (Phila).

[8] Walker SP, Chang SM, Vera-Hernández Marcos, Grantham-McGregor S (2011). Early childhood stimulation benefits adult competence and reduces violent behavior. Pediatrics.

[9] Stingone JA, Sedlar S, Lim S, McVeigh KH (2022). Receipt of early intervention services before age 3 years and performance on third-grade standardized tests among children exposed to lead. JAMA Pediatr.

[10] Choo YY, Yeleswarapu SP, How CH, Agarwal P (2019). Developmental assessment: practice tips for primary care physicians. Singapore Med J.

[11] Council on Children With Disabilities, Section on Developmental Behavioral Pediatrics, Bright Futures Steering Committee, Medical Home Initiatives for Children With Special Needs Project Advisory Committee (2006). Identifying infants and young children with developmental disorders in the medical home: an algorithm for developmental surveillance and screening. Pediatrics.

[12] Eun BL, Kim SW, Kim YK, Kim JW, Moon JS, Park SK, Sung IK, Shin SM, Yoo SM, Eun SH, Lee HK, Lim HT, Chung HJ (2008). Overview of the national health screening program for infant and children. Korean Journal of Pediatrics.

[13] Kim S (2022). Worldwide national intervention of developmental screening programs in infant and early childhood. Clin Exp Pediatr.

[14] Valla L, Wentzel-Larsen T, Hofoss D, Slinning K (2015). Prevalence of suspected developmental delays in early infancy: results from a regional population-based longitudinal study. BMC Pediatr.

[15] Chung HJ, Yang D, Kim G, Kim SK, Kim SW, Kim YK, Kim YA, Kim JS, Kim JK, Kim C, Sung I, Shin SM, Oh KJ, Yoo H, Yu HJ, Lim S, Lee J, Jeong H, Choi J, Kwon J, Eun B (2020). Development of the Korean Developmental Screening Test for Infants and Children (K-DST). Clin Exp Pediatr.

[16] Ghassabian A, Sundaram R, Bell E, Bello SC, Kus C, Yeung E (2016). Gross motor milestones and subsequent development. Pediatrics.

[17] Lopes L, Santos R, Pereira B, Lopes VP (2013). Associations between gross motor coordination and academic achievement in elementary school children. Hum Mov Sci.

[18] Westendorp M, Hartman E, Houwen S, Smith J, Visscher C (2011). The relationship between gross motor skills and academic achievement in children with learning disabilities. Res Dev Disabil.

[19] Lipkin PH, Macias MM, Baer Chen B, Coury D, Gottschlich EA, Hyman SL, Sisk B, Wolfe A, Levy SE (2020). Trends in pediatricians' developmental screening: 2002-2016. Pediatrics.

[20] Suh C, Sohn SY, Kim G, Jung S, Eun B (2016). Single-center experience of the Korean-Developmental Screening Test for infants and children. Korean J Pediatr.

[21] Groos D, Adde L, Aubert S, Boswell L, de Regnier R, Fjørtoft T, Gaebler-Spira D, Haukeland A, Loennecken M, Msall M, Möinichen UI, Pascal A, Peyton C, Ramampiaro H, Schreiber MD, Silberg IE, Songstad NT, Thomas N, Van den Broeck C, Øberg GK, Ihlen EA, Støen R (2022). Development and validation of a deep learning method to predict cerebral palsy from spontaneous movements in infants at high risk. JAMA Netw Open.

[22] Liu X, Chen J, Wang G, Zhang K, Sun J, Ma P, Zhang R (2023). Evaluation of the gross motor abilities of autistic children with a computerised evaluation method. Behaviour & Information Technology.

[23] Suzuki S, Amemiya Y, Sato M (2020). Deep learning assessment of child gross-motor.

[24] Suzuki S, Amemiya Y, Sato M (2021). Skeleton-based visualization of poor body movements in a child's gross-motor assessment using convolutional auto-encoder.

[25] Visser-Bochane MI, Reijneveld SA, Krijnen WP, van der Schans CP, Luinge MR (2020). Identifying milestones in language development for young children ages 1 to 6 years. Acad Pediatr.

[26] Gerber RJ, Wilks T, Erdie-Lalena C (2010). Developmental milestones: motor development. Pediatr Rev.

[27] McWilliams C, Ball SC, Benjamin SE, Hales D, Vaughn A, Ward DS (2009). Best-practice guidelines for physical activity at child care. Pediatrics.

[28] Kim H, Kim J, Jang B, Lee J, Kim J, Lee D, Yang H, Choi Y, Sung M, Kang T, Kim E, Oh Y, Lim J, Hong S, Ahn K, Park C, Kwon S, Park Y (2022). Multiview child motor development dataset for AI-driven assessment of child development. Gigascience.

[29] Papandreou G, Zhu T, Kanazawa N, Toshev A, Tompson J, Bregler C, Murphy K (2017). Towards accurate multi-person pose estimation in the wild.

[30] Ren S, He K, Girshick R, Sun J (2015). Faster R-CNN: towards real-time object detection with region proposal networks. https://papers.nips.cc/paper/2015/hash/14bfa6bb14875e45bba028a21ed38046-Abstract.html.

[31] Sun K, Xiao B, Liu D, Wang J (2019). Deep high-resolution representation learning for human pose estimation.

[32] Duan H, Wang J, Chen K, Lin D PYSKL: towards good practices for skeleton action recognition. arXiv.

[33] Duan H, Zhao Y, Chen K, Lin D, Dai B (2022). Revisiting skeleton-based action recognition.

[34] Liu J, Shahroudy A, Perez M, Wang G, Duan L, Kot AC (2020). NTU RGB+D 120: a large-scale benchmark for 3D human activity understanding. IEEE Trans Pattern Anal Mach Intell.

[35] Chen Y, Zhang Z, Yuan C, Li B, Deng Y, Hu W (2021). Channel-wise topology refinement graph convolution for skeleton-based action recognition.

[36] Chen T, Guestrin C (2016). XGBoost: a scalable tree boosting system.

[37] Selvaraju RR, Cogswell M, Das A, Vedantam R, Parikh D, Batra D (2017). Grad-CAM: visual explanations from deep networks via gradient-based localization.

[38] Pope PE, Kolouri S, Rostami M, Martin CE, Hoffmann H (2019). Explainability methods for graph convolutional neural networks.

[39] Kang MS, Kang D, Kim H (2023). Efficient skeleton-based action recognition via joint-mapping strategies.

[40] Lundberg S, Lee SI (2017). A unified approach to interpreting model predictions. https://papers.nips.cc/paper/2017/hash/8a20a8621978632d76c43dfd28b67767-Abstract.html.

[41] Kim HH, An JI, Park YR (2021). A prediction model for detecting developmental disabilities in preschool-age children through digital biomarker-driven deep learning in serious games: development study. JMIR Serious Games.

[42] Brons A, de Schipper A, Mironcika S, Toussaint H, Schouten B, Bakkes S, Kröse Ben (2021). Assessing children's fine motor skills with sensor-augmented toys: machine learning approach. J Med Internet Res.

[43] Yue R, Tian Z, Du S (2022). Action recognition based on RGB and skeleton data sets: a survey. Neurocomputing.

[44] Moon S, Kim M, Qin Z, Liu Y, Kim D (2023). Anonymization for skeleton action recognition.

[45] Cao Z, Hidalgo G, Simon T, Wei S, Sheikh Y (2021). OpenPose: realtime multi-person 2d pose estimation using part affinity fields. IEEE Trans Pattern Anal Mach Intell.

[46] DigitalHealthcareLab/22ActionRecognitionTool. GitHub.

