# Supplementary material for: Comprehensive Assessment and Early Prediction of Gross Motor Performance in Toddlers With Graph Convolutional Networks–Based Deep Learning: Development and Validation Study
Source: JMIR Form Res. 2024 Feb 21;8:e51996. doi: 10.2196/51996 (PMC10918544; doi:10.2196/51996)
Supplement: Multimedia Appendix 1 [file formative_v8i1e51996_app1.pdf]

Multimedia Appendix 1: Parameters used to train the model.

Parameter	Value
Action evaluation model (**Stage 1)**	
Train : Validation Split	3 : 1
Loss function	Cross entropy loss
Clip Length	80
Augmentation	Flip (Flip Ratio=0.5), Random Scale (Scale Factor=0.1)
Optimizer Strategy	Stochastic Gradient Descent
Initial Learning Rate	$2{\cdot}0 * 10^{-4}$
Momentum	0.9
Weight Decay	$5{\cdot}0 * 10^{-5}$
Learning Rate Policy	Cosine Annealing
Epochs	30
Logging	Every epoch
Number of GPUs	2
Per GPU Train Batch Size	32
GPU Product Name	Quadro RTX 8000
Overall performance prediction model **(Stage 2)**	
Train : Validation Split	3 : 1
Number of Gradient Boosted Trees	100
Minimum Split Loss	0·25

L1 Regularization	$1\cdot5 * 10^{-4}$
L2 Regularization	1·008
Learning Rate	0·3
Subsample Ratio of Columns	0·75
Early Stopping Rounds	20
