# Supplementary material for: Comprehensive Assessment and Early Prediction of Gross Motor Performance in Toddlers With Graph Convolutional Networks–Based Deep Learning: Development and Validation Study
Source: JMIR Form Res. 2024 Feb 21;8:e51996. doi: 10.2196/51996 (PMC10918544; doi:10.2196/51996)

Multimedia Appendix 2: Distribution of top-1 activated joints per frame grouped by body parts. To compare the importance of each body part, we determined the top-1 activated joints that had the highest Grad-Cam values per frame and grouped their frequency by body part. The 17 joints were grouped into the head, left arm, right arm, left leg, and right leg. The distribution of top-1 activated joints were displayed for each label ('bad', 'good' and 'perfect') of four behaviors ('Climb up the stairs', 'Go down the stairs', 'Throw the ball' and 'Stand on one foot'). The results were obtained for the cases when the model correctly predicted the actual labels. GMS (Gross motor skills).

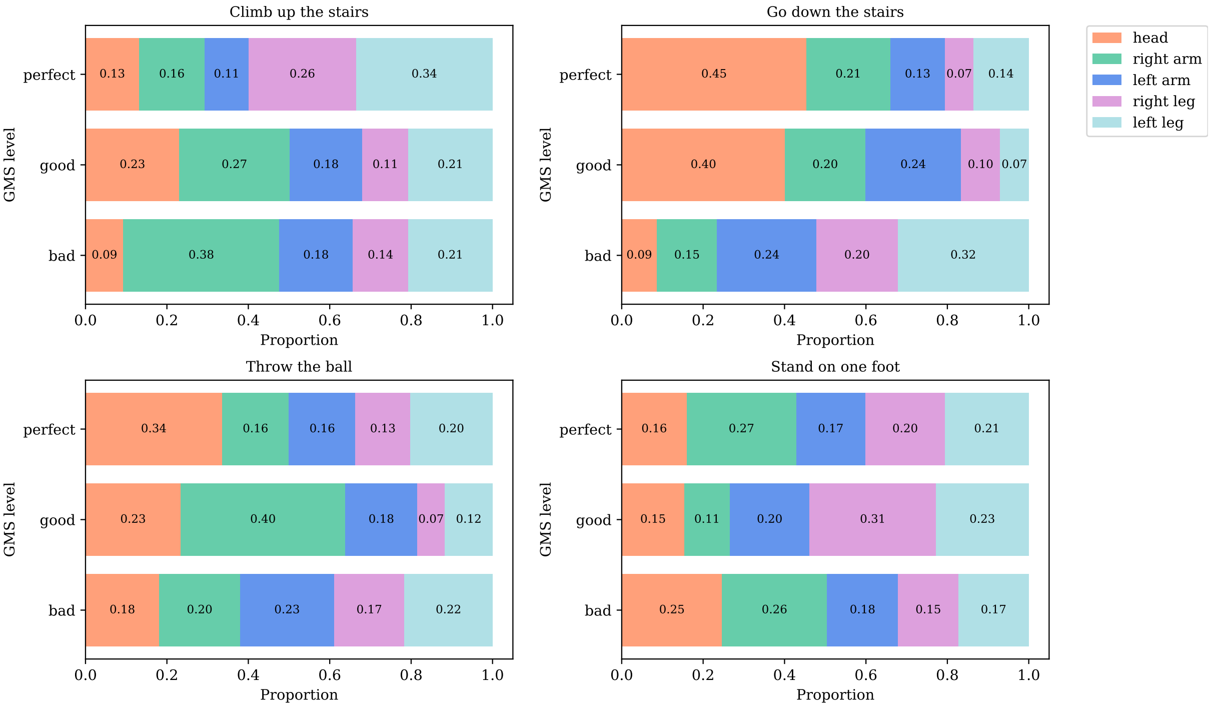

Supplement: Multimedia Appendix 2 [file formative_v8i1e51996_app2.pdf]
